# Prediction of Pancreatic Islet Yield After Pancreatectomy Using Optical Coherence Elastography

**DOI:** 10.3390/diagnostics16020329

**Published:** 2026-01-20

**Authors:** Ekaterina Gubarkova, Ekaterina Vasilchikova, Arseniy Potapov, Denis Kuchin, Polina Ermakova, Julia Tselousova, Anastasia Anina, Liya Lugovaya, Marina Sirotkina, Natalia Gladkova, Aleksandra Kashina, Vladimir Zagainov

**Affiliations:** 1 Institute of Experimental Oncology and Biomedical Technologies, Privolzhsky Research Medical University, 603950 Nizhny Novgorod, Russia; 2 Department of General, Operative Surgery and Topographic Anatomy Named After A.I. Kozhevnikov, Privolzhsky Research Medical University, 603950 Nizhny Novgorod, Russia; 3 Research Institute of Clinical Oncology “Nizhny Novgorod Regional Clinical Oncological Dispensary”, 603126 Nizhny Novgorod, Russia; 4 Department of Endocrinology and Internal Medicine, Privolzhsky Research Medical University, 603950 Nizhny Novgorod, Russia; 5 Department of Faculty Surgery and Transplantation, Privolzhsky Research Medical University, 603950 Nizhny Novgorod, Russia

**Keywords:** optical coherence elastography (OCE), stiffness, pancreatic parenchyma, pancreatic ductal adenocarcinoma (PDAC), islets of Langerhans, pancreatectomy

## Abstract

Intraoperative assessment of pancreatic quality, followed by sampling for the potential isolation of Langerhans islets for subsequent autotransplantation, is currently a key component of post-total pancreatectomy diabetes mellitus treatment. The aim of this study was to quantitatively evaluate pancreatic parenchymal stiffness using optical coherence elastography (OCE) imaging, and to investigate the utility of the OCE method as a potential indicator of islet yield after pancreatectomy. A total of 41 freshly excised human pancreatic specimens, containing pancreatic ductal adenocarcinoma (PDAC) and surrounding non-tumorous tissues post-pancreatectomy, were studied. In this research, the stiffness (Young’s modulus, kPa) and its color-coded 2D distribution were calculated for various pancreatic samples using compression OCE. Stiffness values were compared between intact pancreatic parenchyma (islet-poor and islet-rich) and pancreatic lesion groups (parenchymal fibrosis and/or PDAC invasion). The data were confirmed by histological analysis. In addition, the measured stiffness values for various morphological groups of the pancreatic samples were compared with the number of isolated islets obtained from pancreatic samples after collagenase treatment. The study demonstrated that OCE can effectively distinguish areas of pancreatic lesions and identify intact pancreatic parenchyma containing Langerhans islets. A highly significant increase in mean stiffness (*p* < 0.0001) was observed in postoperative pancreatic samples exhibiting signs of parenchymal fibrosis or PDAC invasion compared to unaffected, intact pancreatic parenchyma. For the first time, a relationship between stiffness values and the number of isolated pancreatic islets was demonstrated; in particular, the number of isolated islets significantly decreased (≤110 pcs/g) in samples exhibiting stiffness values above 150 kPa and below 75 kPa. The optimal stiffness range for the efficient isolation of islets (≥120 pcs/g) from pancreatic tissue was identified as 75–150 kPa. The study introduces a novel approach for rapid and objective intraoperative assessment of pancreatic tissue quality using real-time OCE data. This technique facilitates the identification of regions affected by pancreatic lesions and supports the selection of intact pancreatic parenchyma, potentially enhancing the accuracy of Langerhans islet yield predictions during surgical resection.

**Figure 1:**
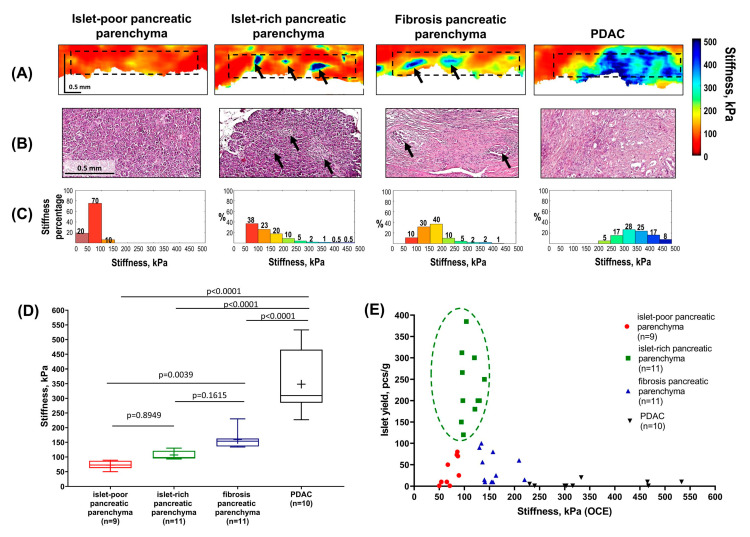
**Quantification of the stiffness of intact pancreatic parenchyma (islet-poor and islet-rich) and pancreatic lesion groups (parenchymal fibrosis and/or PDAC invasion) using compression optical coherence elastography (OCE) and comparison with islet yield from collagenase-treated pancreatic tissue.** Total pancreatectomy in patients with pancreatic pathology is associated with low postoperative mortality (approximately 5%) [1], but it results in severe endocrine insufficiency and significant metabolic complications, including brittle diabetes mellitus that is difficult to manage [2]. To prevent postoperative diabetes and its sequelae, intraportal islet autotransplantation is performed after total pancreatectomy. This procedure reduces dependence on long-term insulin therapy and improves glycemic control [3]. Accurate quantification of pancreatic tissue quality is essential for evaluating the feasibility and efficiency of islet isolation from post-pancreatectomy specimens [4] and may be influenced by the extent and pattern of pancreatic morphological damage. The presence of fibrosis of varying severity, including post-chemotherapy fibrosis, fibrocystic changes, inflammatory processes, or tumor invasion in patients with pancreatic cancer, all of which are associated with increased tissue stiffness, may adversely affect the isolation of viable, functional islets of Langerhans [4,5,6,7]. From this perspective, pancreatic tissue obtained from patients with pancreatic ductal adenocarcinoma can be regarded as a clinically relevant, fibrosis-dominant model, enabling systematic investigation of stiffness-related alterations and their impact on islet isolation under controlled conditions. Until now, pancreatic tissue characterization has relied primarily on biopsy with histological analysis or medical imaging modalities such as ultrasound, computed tomography, or magnetic resonance imaging [8]. However, these methods can be labor-intensive or lack sufficient spatial resolution to effectively visualize the microstructural features of pancreatic tissue and the presence of islets. Consequently, there is a need for novel intraoperative imaging techniques, and optical coherence elastography (OCE) represents a promising candidate. The study was carried out on 41 freshly excised human pancreatic specimens obtained from 41 patients (ages 38–83) with stage I or II (T1–2 N0–1 M0) pancreatic ductal adenocarcinomas (PDACs) after pancreatectomy. Nine patients developed type 2 diabetes mellitus before their cancer diagnosis. Eleven patients had received neoadjuvant chemotherapy with FOLFIRINOX prior to resection. For each patient, pancreatic parenchyma samples were obtained at a distance of at least 10 mm from the tumor. The specimens ranged in size from 0.5 × 1.0 × 0.5 cm^3^ to 1.0 × 2.0 × 0.5 cm^3^. (**A**) OCE images of the four morphological types of pancreatic specimens were acquired using a 20 kHz spectral-domain OCT system with a central wavelength of 1.3 µm [9,10,11] combined with the phase-sensitive compression OCE mode for assessing the biomechanical properties of the tissues [12]. For OCE image reconstruction and evaluation of the absolute stiffness (Young’s modulus, kPa) of the pancreatic parenchyma, the specimen was compressed by the OCT probe through an intermediate layer of reference silicone with 100 kPa [12,13,14]. OCE estimates were obtained for a standardized stress (2 ± 1 kPa in this study) to account for the fact that human pancreas is a nonlinear viscoelastic soft tissue [15,16]. It was established that homogeneous low stiffness values (<100 kPa) characterized islet-poor pancreatic parenchyma. In contrast, heterogeneous distribution of low stiffness values with isolated areas of increased stiffness (>250 kPa) was observed in islet-rich (black arrows) pancreatic parenchyma. In cases where fibrosis pancreatic parenchyma was present due to chronic pancreatitis, stiffness values increased (>100 kPa) compared to intact pancreatic parenchyma. When regions of a PDAC were present, stiffness values significantly exceeded those of intact and fibrosis pancreatic parenchyma, reaching over 400 kPa in extensive high-stiffness areas. As a result, OCE with a spatial resolution of 30–50 µm enables identification of pancreatic islets at the histological scale based on localized high-stiffness inclusions within the surrounding softer acinar tissue and allows estimation of islet abundance, which may serve as a potential indicator for successful islet isolation. Additionally, OCE enables the detection of acinar tissue areas without islets, as well as significantly harder regions of PDAC and fibrotic pancreatic parenchyma, which could lead to a reduction in tissue quality and a low yield of islets. (**B**) After OCE imaging, histological sections were prepared; their planes coincided with the OCE images and stained with hematoxylin and eosin (H&E). Histological analysis verified the OCE data and demonstrated the pancreatic samples of preserved pancreatic parenchyma, both islet-poor and islet-rich (black arrows), as well as parenchyma with PDAC and fibrous parenchyma with reduced acinar tissue. Islet-poor pancreatic parenchyma is a normal variant and is likely related to the anatomical site of sampling. The presence of fibrous parenchyma is a common observation in PDAC and is a consequence of block of the main pancreatic duct, chronic pancreatitis or effects of chemotherapy [4,17,18]. (**C**) The next step was to obtain and analyze the “stiffness spectra” for the pancreatic specimens, i.e., histograms showing percentages of pixels with different Young’s modulus values within a chosen region of interest (~350 × 3200 μm, outlined by a black dashed rectangle in (**A**)). The analysis of normalized histograms for the four morphological groups of the pancreas show demonstrates a significant shift in the stiffness distribution: in islet-poor pancreatic parenchyma, 90% of pixels had extremely low stiffness values below 100 kPa. Conversely, the “stiffness spectra” for islet-rich pancreatic parenchyma, fibrous pancreatic parenchyma and PDAC shift toward higher stiffness values. Notably, pancreatic parenchyma with islets exhibits the most heterogeneous distribution of stiffness, with a clear decrease in the proportion of the softest tissue components (to 38%) and the presence of a small fraction of pixels (~9%) measuring over 250 kPa and corresponding morphologically to the presence of pancreatic islets. In cases of fibrotic parenchyma, the proportion of the softest tissue components decreases to 10%, whereas intermediate average stiffness values (~80%) predominate. For PDAC, the proportion of stiffer areas (>250 kPa) is highest, exceeding 95%. In comparison with a mean stiffness shown in the next panel (**D**), such stiffness distributions give more detailed representation about the difference between various pancreatic samples. (**D**) The distribution diagram of mean stiffness values for pancreatic samples —both non-pathological and with signs of damage—showed a statistically significant increase in stiffness in postoperative pancreatic samples exhibiting fibrosis due to chronic pancreatitis or tumor invasion, compared to intact pancreatic parenchyma. In this study, to provide more accurate identification of morphological pancreatic structures in the OCE images, we scanned a large specimen field and then analyzed the area consisting of 3–4 stitched OCE images. For each patient, three OCE images were quantitatively analyzed to better capture the consistency of stiffness measurements, taking into account morphological heterogeneity and the focal nature of certain pathological changes. Center line in the boxes—median; “+”—mean values; box limits—25th and 75th percentiles; whiskers—minimum and maximum values within the 1.5× interquartile range of the first and third quartile. Segment indicates a statistically significant difference between the study groups (the Mann–Whitney U-test for multiple comparison were used to detect significant differences in numerical data between independent groups), where p is the magnitude of the statistical significance of the differences between states of pancreatic tissue and n is the number of examined pancreatic specimens for each group. (**E**) Distribution of the number of isolated Langerhans islets relative to the stiffness values in pancreatic samples. Islet isolation was performed according to a previously developed method [5]. Briefly, pancreatic tissue samples were subjected to enzymatic digestion using collagenase NB1 (3 mg/mL) and neutral protease (0.8 mg/mL). Digestion was performed in two stages and lasted 20–35 min in total (20–30 min followed by an additional 5–10 min). After digestion, enzymatic activity was monitored by dithizone staining to assess the release of islets from acinar tissue. If more than 50% of islets remained attached, a short additional incubation with collagenase was performed. Enzymatic digestion was stopped by dilution with cold wash solution, followed by repeated centrifugation and washing steps at 4 °C. The digested tissue was then filtered, and islets were purified using a discontinuous Ficoll density gradient. As a result, it was demonstrated that the success of islet isolation depends on the quality of the pancreatic parenchyma sample. A high yield of islets (≥120 pcs/g) was observed in samples with medium stiffness (75–150 kPa), corresponding morphologically to acinar tissue rich in islets (green dotted box in (**E**)). The number of isolated islets decreased (≤110 pcs/g) in samples with high stiffness (>150 kPa), indicative of fibrosis or tumor invasion, and also in samples with low stiffness (<70 kPa), which morphologically corresponded to acinar tissue devoid of islets. Furthermore, both previous studies and our preliminary observations indicate that hyperglycemia and diabetes may arise secondary to pancreatic cancer progression or neoadjuvant therapy [19] and are associated with reduced islet yield and altered islet quality [20,21]. Importantly, pancreatic tissue adjacent to PDAC exhibits structural and biomechanical alterations, including increased extracellular matrix deposition and reduced enzymatic digestibility, that overlap with changes reported in chronic pancreatitis and other fibrotic pancreatic conditions [8,22,23,24]. These pathological changes can potentially impair the isolation process and affect the quality of islets for subsequent transplantation. This aligns with previous studies showing that histologically confirmed chronic pancreatitis results in poor islet isolation [25], and that fibrotic tissue is more resistant to collagenase digestion [26]. Along with the promising results, several limitations of the OCE method should be acknowledged, including limited penetration depth (approximately 2 mm in air) and relatively small tissue scanning fields (up to 4 mm). In addition, in the present study OCE enabled detection of the presence and spatial distribution of pancreatic islets but did not allow for cellular-level characterization. The resolution of OCE images (typically 30–50 μm) does not reach that of histological analysis, therefore minor discrepancies in the size of individual structural components of the tissue may occur. Owing to the lack of cellular resolution, the exact size of individual islets and their total number within pancreatic tissue could not be determined. Nevertheless, the OCE data presented above can already be used to select “soft” intact pancreatic parenchyma as a potential indicator of islet yield. The obtained results suggest that OCE-based assessment of pancreatic tissue quality using stiffness as a prognostic indicator of islet yield may also be relevant for selected non-oncologic pancreatic conditions characterized by fibrosis. Therefore, for the first time, the potential of the OCE method was demonstrated for an objective, quantitative assessment of post-operative pancreatic tissue samples based on the level and distribution pattern of stiffness (Young’s modulus) values in kPa, with the aim of selecting samples for subsequent isolation of Langerhans islets. Furthermore, we believe that the results reported here represent a baseline in the use of this technique and are a first step towards establishing its use in a clinical setting. In the future, the use of C-OCE to measure the elastic properties (stiffness) of the pancreas during surgery may be the next step forward for quantitative assessment of pancreatic quality. Using such a technique, pancreas could be examined in vivo intraoperatively—before resection, during resection in the resection bed, and after resection on the resected specimen.

## Data Availability

The data presented in this study are available on request from the corresponding author due to privacy or ethical restrictions.

## Abbreviations

The following abbreviations are used in this manuscript:

OCE	Optical coherence elastography
PDAC	Pancreatic ductal adenocarcinoma
OCT	Optical coherence tomography
H&E	Hematoxylin and eosin
